# Relationship between fear of falling and fall risk among older patients with stroke: a structural equation modeling

**DOI:** 10.1186/s12877-023-04298-y

**Published:** 2023-10-11

**Authors:** Yuanyuan Chen, Hui Du, Mi Song, Ting Liu, Pei Ge, Yue Xu, Hongying Pi

**Affiliations:** 1 Medical School of Chinese PLA, Beijing, People’s Republic of China; 2 Department of Cardiology, Second Medical Center, Chinese PLA General Hospital, Beijing, People’s Republic of China; 3 Medical Service Training Center, Chinese PLA General Hospital, No. 28 Fuxing Road, Haidian District, 100853 Beijing, People’s Republic of China

**Keywords:** Stroke, Fear of falling, Accidental fall, Depression, Postural balance

## Abstract

**Background:**

With reduced balance and mobility, older patients with stroke are more susceptible to fear of falling (FOF). A maladaptive form of FOF can cause excessive activity restriction, poor balance, and recurrent falls, forming a self-reinforcing vicious cycle. This study applied and adapted the FOF model to investigate the interaction between FOF and fall risk in older stroke patients.

**Methods:**

A cross-sectional study was conducted among 302 older stroke patients aged 60 and over. All participants were invited to complete the FOF, fall risk, physical activity, and balance tests, which were measured by the Falls Efficacy Scale International (FES-I), Self-Rated Fall Risk Questionnaire (FRQ), the long-form International Physical Activity Questionnaire (IPAQ-LF) and the Four-Stage Balance Test (FSBT) respectively. Data were analyzed using structural equation modeling.

**Results:**

The mean age of the respondents was 68.62 ± 7.62 years; 8.94% reported a high level of FOF, and 18.21% reported a moderate level of FOF. The structural equation model showed that FOF was directly associated with fall risk (*β*=-0.38, *p* < 0.001), and was indirectly associated with fall risk via physical activity (*β*=-0.075, *p* < 0.05) and balance ability (*β*=-0.123, *p* < 0.05). Depression (*β*=-0.47, *p* < 0.001), fall history (*β*=-0.13, *p* < 0.05), and female sex (*β*=-0.16, *p* < 0.05) affected FOF, while anxiety was not associated with FOF.

**Conclusions:**

The increased risk of falling in older stroke patients results from a maladaptive FOF affected by depression, fall history, poor balance ability, and limited physical activity. Our results suggest that greater attention should be paid to FOF during stroke recovery and fall prevention. A multifaced intervention program encompassing physiological and psychological factors should be designed to address FOF and prevent falls.

## Background

The relationship between the fear of falling (FOF) and the risk of falling has drawn broad attention [1]. FOF may serve a useful purpose in heightening self-awareness about adaptive strategies to prevent a future fall [2]. However, in some cases, FOF can be maladaptive in that it stimulates a vicious cycle of deconditioning, thereby making the person more susceptible to a future fall [3]. Even though recent research questioned the extent of the prediction of FOF [4], this negative relationship remains for those with balance/gait impairments [5]. For older patients with stroke, their impaired balance and sensory systems contribute to the high incidence of FOF and falls [6]. Studies show that 32–66% of stroke patients have FOF [7, 8], and 26–73% suffer at least one fall 6 months post-stroke [9]. A high level of FOF and fall incidence can limit rehabilitation exercise, reduce mobility and independence ability, increasing mortality [7]. Studies suggested a mutual relationship between FOF and fall risk. Falls can cause FOF, and then FOF can cause increased fall risk. The two outcomes may be related to other shared risk factors and not causally related [10], and it is still unclear whether they are directly or indirectly linked.

**Fig. 1:**
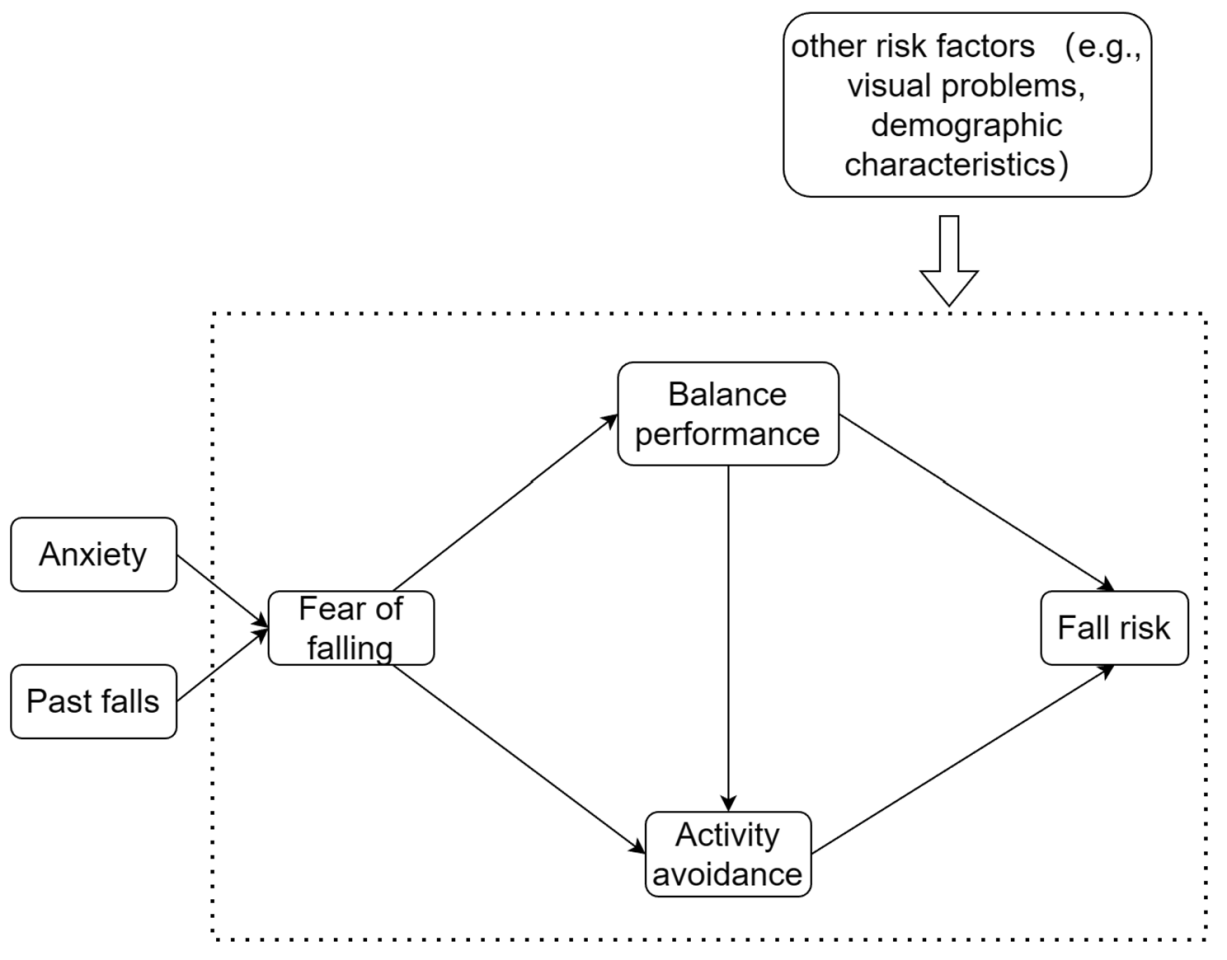
Simplified theoretical framework depicting the way from fear of falling towards fall risk. Adapted from Hadjistavropoulos (2011)

The Fear of Falling Model [11] proposes a way to understand the formation of FOF and how it reinforces fall risk from the perspective of fear-avoidance theory. According to the original model (Fig. 1), FOF is caused by both physiological factors such as fall history and psychological factors such as mood/temperament; these factors cause excessive activity restriction and poor balance, further increasing the risk of falling. This original model forms a self-reinforcing vicious cycle that offers a theoretical basis for studying the relationship between FOF and fall risk. It has been validated in patients with Parkinson’s disease [12]; whether this model applies to older stroke patients and what role FOF plays in the model remains unknown [13].

Several investigations have been conducted regarding the risk factors for FOF and its relationship with fall risk in stroke patients [14–19]; they suggested that FOF is a critical indicator of falling; however, these studies investigating the connection between FOF and fall risk lack theoretical underpinnings explaining how physiological and psychological factors induce FOF and fall risk, and they are not supported by detailed assessments of relevant constructs [15, 18]. Theory-based and extensive investigations are needed to develop a further understanding of what FOF means to older stroke patients. Therefore, this cross-sectional study aimed to learn the risk factors of FOF and its relationship with fall risk by building a FOF model for older stroke patients; this might provide new insights in opportunities for the prevention and management of FOF and fall risk in older patients with stroke.

## Methods

### Study population and data collection

This cross-sectional study was conducted in a hospital in the capital city. With a patient volume of over 2 thousand every day, this hospital receives diverse and sufficiently large patients from the whole country. This enables researchers to recruit targeted populations and prescreen them based on the criteria. A convenience sampling method was adopted. Participants were enrolled from March to September 2022. Inclusion criteria: 60 years and older; well-documented stroke (clinical presentation and MRI scan of the brain, ischemic or hemorrhagic type) and more than 6 months post-stroke; ability to walk independently or using a walker. Exclusion criteria: severe cognitive impairment as defined by a score of less than 20 on the Mini-Mental State Examination (MMSE) [20]; too medically unstable to participate; neurological disease other than stroke; severe mental illness requiring oral medication; not Chinese-speaking. All the participants were informed about the purpose of the study and provided informed consent before the survey. They underwent a questionnaire-based face-to-face survey delivered by trained researchers. The survey took 30–40 min to complete. The estimation criteria proposed by Hair [21] were followed to ensure a sufficient sample size for structural equation modeling. Considering the number of indicators, an estimated power > 80%, and α = 0.05, the estimated sample size was 300. Researchers approached 309 eligible subjects, 5 of whom refused, and 2 dropped out. Finally, complete data from 302 participants were evaluated, which is sufficient for statistical analysis. The present research received ethical approval from the ethics committee of the Chinese PLA General Hospital (S2021-077-01).

### The hypothesized model with fear of falling

**Fig. 2:**
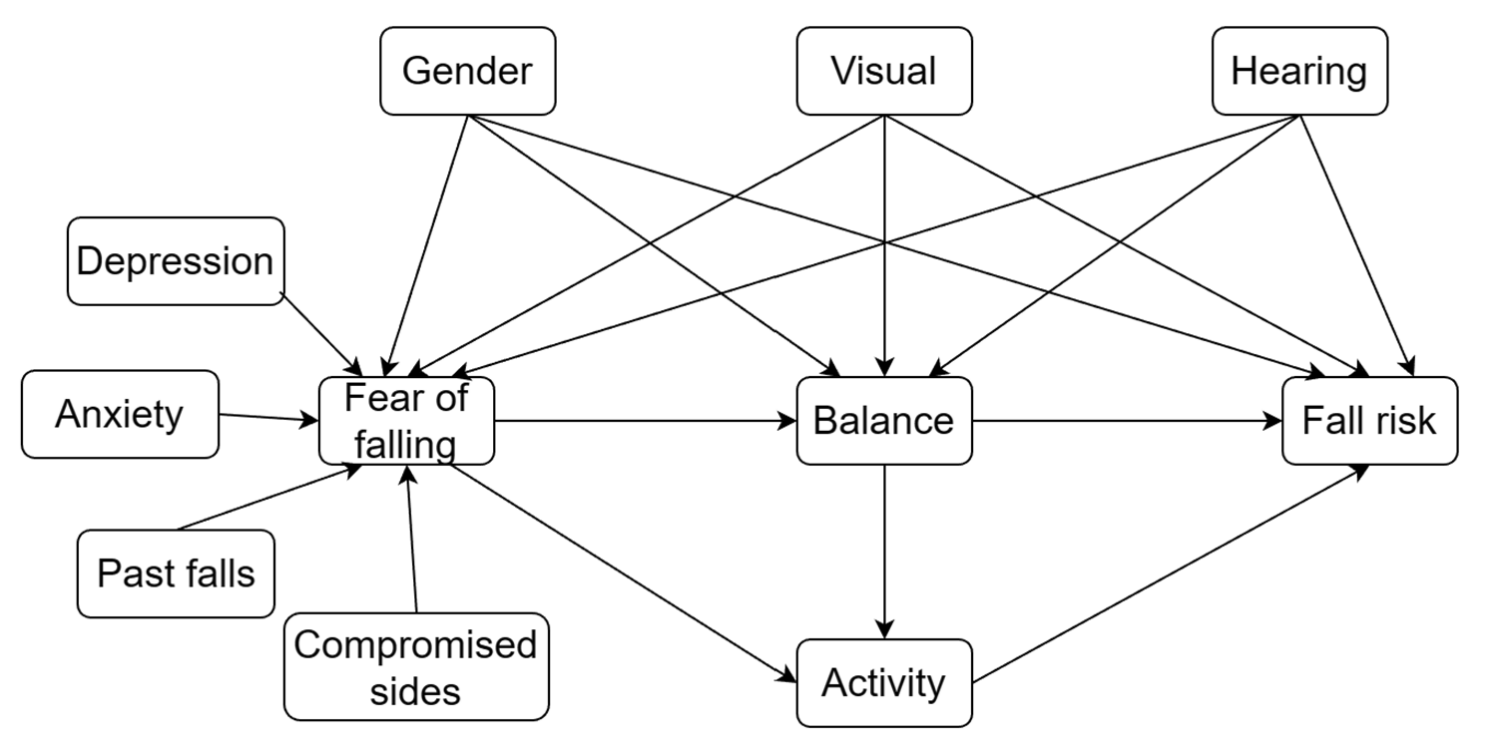
The hypothesized model with fear of falling and the extension of hearing problems, compromised sides, and depression

The original model by Hadjistavropoulos in 2011 (Fig. 1) explained the impact of FOF on fall risk, the mediating role of balance ability, and self-imposed activity restriction. Based on the original model, the hypothesized model was built. To enhance the prediction of the constructs, relevant aspects were added to the model suggested by previous research. First, we embedded depression into the model extension; previous work found that depression as part of the mental state could significantly increase the risk of FOF in patients with stroke [16]. Second, the difference in the compromised sides (e.g., right, left, and bilateral) might affect FOF [22]. Third, the analysis suggested that sensory difficulties such as hearing problems were related to FOF, fall risk, and balance performance [23, 24]. The hypothesized interactions inside the model are presented in Fig. 2.

### Variables and measures

Demographic information included: age, gender, height and weight. Clinical data included: the date of stroke, compromised sides (right or left or bilateral) [22], hearing impairment and visual impairment. Hearing impairment was measured by one single question, “Do you feel you have a hearing loss?” the response options were yes or no [23, 25]. Visual impairment was assessed by the question, “How well can you see from a distance?” the response options were dichotomized into “Well” and “Not well.” [23]. Body Mass Index (BMI) was calculated by weight in kilograms divided by the square of height in meters; BMI ≥ 28.0 kg/m^2^ indicates obesity [26]. Fall history was represented by the occurrence of at least one fall in the past year [10]. The definition of fall is “an unexpected event in which the individual comes to rest on a lower level such as the ground, floor, or steps.” [27].

### Fear of falling

Fear of falling is a related but different construct from balance confidence and balance self-efficacy [28], whereas they are all psychological indicators of balance-related confidence. FOF was assessed using methods including the Falls Efficacy Scale International (FES-I) [29], the Short Falls Efficacy Scale International (Short FES-I) [30], Iconographical Falls Efficacy Scale (Icon-FES) [31], the Survey of Activities and Fear of Falling in the Elderly (SAFE) [32], a visual analogue scale [33], or a dichotomous variable (yes or no) [34]. Balance confidence is usually measured by the Activities-Specific Balance Confidence Scale (ABC) [35]. FES-I is the most commonly used tool for measuring FOF [36]; it was adapted from the Falls Efficacy Scale in 2005 [37] and was validated in stroke patients [38]. FES-I contains 16 activities of both primary and demanding, physical and social [37]. For each item, there are four response items: “not all confident”, “somewhat confident”, “fairly confident”, and “very confident”, labelled as 1 to 4. The sum of the score ranges from 16 to 64 points, with a score of 16 to 19 indicating a high level of FOF, 20 to 27 indicating a moderate level of FOF, and 28 to 64 indicating a low level of FOF [39].

### Physical activity behaviour

The long-form International Physical Activity Questionnaire (IPAQ-LF) was used to assess physical activity behaviour [40]. The IPAQ-LF consists of activities with low, moderate and high intensity; the activities’ domains contain leisure time physical activity, domestic and gardening activities, work-related physical activity, and transport-related physical activity. The 27 questions reflect the previous seven days’ activities; total physical activity of metabolic equivalent (MET)-minutes/week was calculated to describe the amount of exercise.

### Fall risk

The Self-Rated Fall Risk Questionnaire (FRQ) serves as a fall risk screening tool of the STEADI algorithm [41]; it comprises 12 questions related to the real-life risk factors of falls. The score of the labels can be 0, 1 or 2; a score of 4 and over shows that the patient is at risk [42].

### Balance performance

The Four-Stage Balance Test (FSBT) is part of the Centers for Disease Control and Prevention (CDC)-recommended STEADI test protocol for balance function [43]. The patient was instructed to maintain 4 challenging positions without any assisting device (e.g., crutch or stick); each successive position became more difficult to hold. The position was changed every 10s, and the test ended when the subject could no longer maintain a position. Being unable to hold the tandem stance (task number 3) for 10s indicates poor balance function [44].

### Depression

Depression was measured with the 15-item Geriatric Depression Scale (GDS-15); it is a suitable tool to detect depressive disorder for stroke patients [45], the total score ranges from 0 to 15, and a score of 8 and over indicates the presence of depression.

### Anxiety

The 7-item Generalized Anxiety Disorder scale (GAD-7) was used to measure anxiety. It has been used in stroke patients as part of mental health outcomes [46]. The range of scores is 0 to 21; a score of 5 to 9 indicates a low level of anxiety, 10 to 14 indicates a medium level of anxiety, and 15 to 21 indicates a high level of anxiety [47].

### Data analysis

The IBM SPSS Statistics 26 was used to record and process data. The statistics were stratified by the FOF category. A one-way analysis of variance (ANOVA) test for continuous variables (height, mass, BMI, FES-I, FRQ, FSBT, GAD-7, GDS-15, IPAQ-LF) and chi-squared tests for categorical variables (all other variables) were conducted to compare the low, moderate and high FOF groups with each other on the variables. A *P*-value of < 0.05 was considered significant. IBM SPSS Amos 26 Graphics was employed to conduct structural equation modeling. We used the maximum likelihood estimation method to test the model. Cutoff points for the root mean square error of approximation (RMSEA ≤ 0.08 with a confidence interval of 95%), the comparative fit index (CFI ≥ 0.90), as well as the Goodness of fit index (GFI ≥ 0.90) were used to assess proper fitness [48].

## Results

### Descriptive

The mean age of the participants was 68.62 (SD 7.62) years; 38.4% were female. The mean body mass index was 24.44 (SD 3.29). 8.94% (*n* = 27) indicated high FOF, and 18.21% *(n* = 55) reported moderate FOF. Further, 33.8% (*n* = 102) had experienced at least one fall in the past year. Table 1 shows the comparisons of the participant characteristics among the low (*n* = 220), moderate (*n* = 55), and high (*n* = 27) levels of FOF groups. Gender, age, marital status, fall history, visual and hearing problems, fall risk, activity, balance, depression, and anxiety were prominent in participants with different levels of FOF.

**Table 1:** Participant characteristics (N = 302)

Variable	Mean ± SD or no. (%)								*P* value
	Fear of falling	Fear of falling			Fear of falling				
	Low (n = 220)	Moderate (n = 55)			High (n = 27)				
**Gender**									0.045
Male	144(77.42)	26(13.98)			16(8.60)				
Female	76(65.52)	29(25.00)			11(9.48)				
**Age**									0.002
60–69	148(80.00)	26(14.05)			11(5.95)				
70–79	49(62.03)	22(27.85)			8(10.13)				
80 and above	23(60.53)	7(18.42)			8(21.05)				
**Height,m**	1.67 ± 0.07	1.65 ± 0.08			1.66 ± 0.08				0.080
**Mass, kg**	69.18 ± 10.42	66.59 ± 10.49			68.63 ± 11.71				0.268
**BMI, kg/m2**	24.75 ± 3.10	24.63 ± 3.82			25.02 ± 3.70				0.881
**Education**									0.128
Elementary school or less	26(68.42)	5(13.16)			7(18.42)				
Middle school	123(72.35)	36(21.18)			11(6.47)				
Junior college and above	71(75.53)	14(14.89)			9(9.57)				
**Marital status**									0.035
Married	201(75.28)	46(17.23)			20(7.49)				
Divorced	2(50.00)	1(25.00)			1(25.00)				
Widowed	17(54.84)	8(25.81)			6(19.35)				
**Compromised side**									0.970
Left	54(72.00)	14(18.67)			7(9.33)				
Right	44(69.84)	13(20.63)			6(9.52)				
Bilateral	122(74.39)	28(17.07)			14(8.54)				
**Fall history**									0.001
Yes	62(60.78)	24(23.53)			16(15.69)				
No	158(79.00)	31(15.50)			11(5.50)				
**Visual problems**									0.048
Yes	85(69.11)	21(17.07)			17(13.82)				
No	135(75.42)	34(18.99)			10(5.59)				
**Hearing problems**									0.048
Yes	70(67.96)	18(17.48)			15(14.56)				
No	150(75.38)	37(18.59)			12(6.03)				
**FES-I, points**	45.13 ± 9.80	23.27 ± 2.56			18.22 ± 0.93				0.000
**FRQ, points**	5.13 ± 3.04	8.45 ± 2.79			9.96 ± 2.22				0.000
**FSBT, s**	51.77 ± 21.19	24.25 ± 23.29			12.76 ± 22.70				0.000
**GAD-7, points**	5.65 ± 3.25	7.60 ± 3.63			8.11 ± 3.25				0.000
**GDS-15, points**	3.93 ± 2.67	5.72 ± 2.88			7.59 ± 3.29				0.000
**IPAQ-LF, MET·min/wk**	4217.73 ± 2967.48	2101.78 ± 2693.68			1069.96 ± 1978.18				0.000

Abbreviation: BMI, body mass index; FES-I, the Falls Efficacy Scale—International; FRQ, the Self-Rated Fall Risk Questionnaire; FSBT, the Four-Stage Balance Test; GAD-7, the 7-item Generalized Anxiety Disorder scale; GDS-15, the 15-item Geriatric Depression Scale; IPAQ-LF, the long-form International Physical Activity Questionnaire; MET, metabolic equivalent; SD, standard deviation

### Model effects

**Fig. 3:**
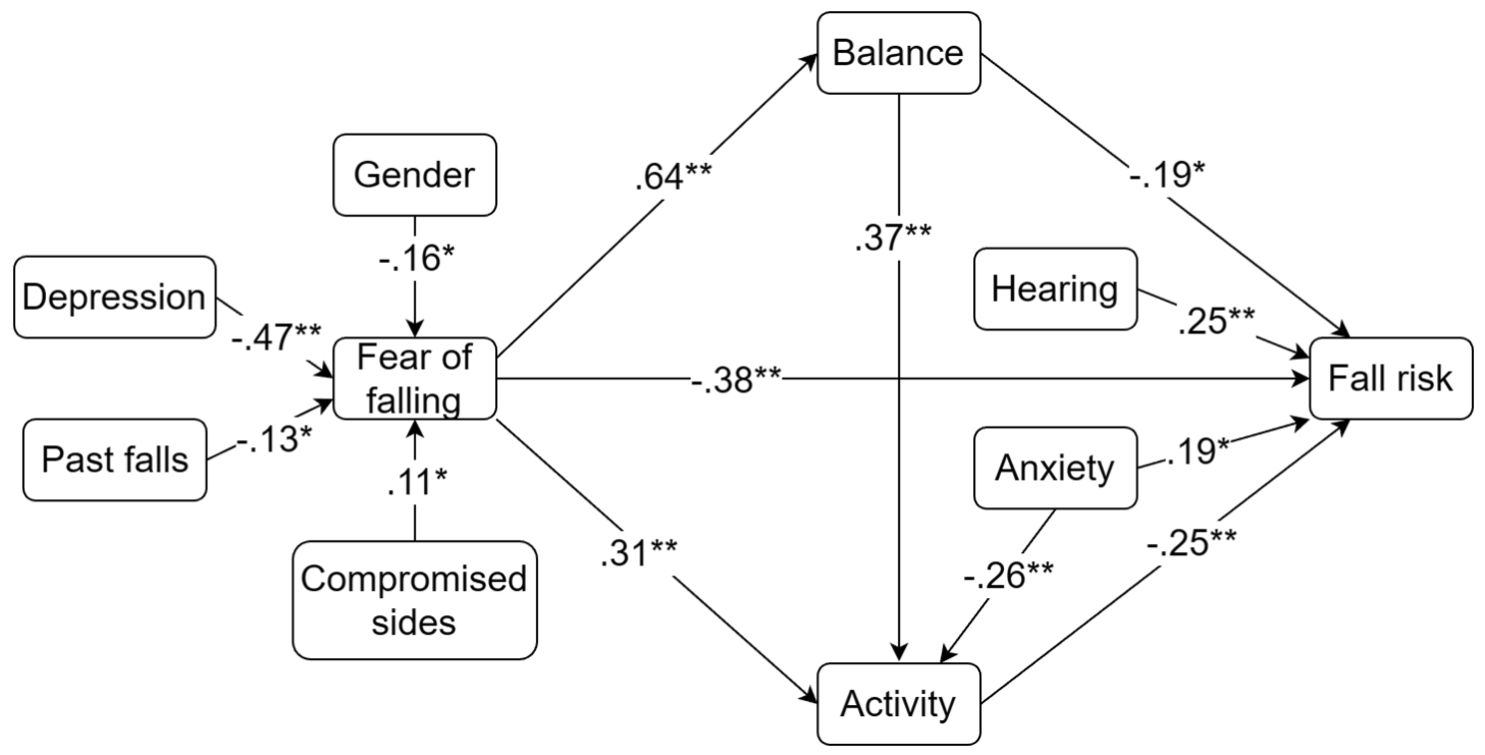
The final model from the structural equation modeling predicts older stroke patients’ fear of falling and fall risk using the fear of falling model. Only significant paths are shown. *p < 0.05, **p < 0.001

The structural equation modeling was processed with Amos, and all items for each construct demonstrated reliable factor loadings. The model produced adequate fit (χ^2^ = 255.34, df = 244, *p* < 0.001, RMSEA = 0.01, CFI = 1.00, GFI = 0.94), and all parameters indicated goodness of fit. The final model is illustrated in Fig. 3.

### Factors associated with fall risk

As hypothesized, FOF (β=-0.38, *p* < 0.001), activity (β=-0.25, *p* < 0.001), balance ability (β=-0.19, *p* < 0.05), hearing problems (β = 0.25, *p* < 0.001) and anxiety (β = 0.19, *p* < 0.05) were associated with fall risk. Analysis for indirect effects found that physical activity behaviour (β=-0.075, *p* < 0.05, 95% CI -0.154 to -0.023), balance ability (β=-0.123, *p* < 0.05, 95% CI -0.213 to -0.007) acted as mediators separately between FOF and fall risk. Physical activity and balance ability could play a chain mediating role between FOF and fall risk (β=-0.059, *p* < 0.05, 95% CI -0.151 to -0.020). Unlike the hypothesis, visual problems and gender were unrelated to fall risk (*p* > 0.05).

### Factors associated with FOF

According to the final model, depression (β=-0.47, *p* < 0.001), fall history (β=-0.13, *p* < 0.05), female sex (β=-0.16, *p* < 0.05), left or right compromised side (β = 0.11, *p* < 0.05) predicted worse FOF. Unlike the hypothesis, anxiety, visual, and hearing problems were not directly associated with FOF (*p* > 0.05).

### Factors associated with balance ability and activity behaviour

FOF (β = 0.64, *p* < 0.001) was the only construct that predicted balance ability. Gender, hearing or visual problems were unrelated to balance ability (*p* > 0.05). Good balance ability (β = 0.37, *p* < 0.001) was associated with active activity behaviour; anxiety (β=-0.26, *p* < 0.001) and FOF (β = 0.31, *p* < 0.001) were negative predictors of activity behaviour.

## Discussion

A model of FOF for older patients with stroke was developed in the study. It illustrated the influence of physiological and psychological factors on the risk of falling. The model initially confirmed the maladaptive form of FOF and indicated the following main findings: firstly, FOF directly affected fall risk or indirectly affected fall risk through balance ability and activity behaviour pathways. Numerous studies proved that FOF interferes with daily activity, affecting individuals’ physical well-being [2, 49]. FOF patients tend to adopt sedentary lifestyles, reducing both physical and social activities as a way to avoid falls; this can prevent falls in the short period, but in the long run, this kind of self-imposed reduction of daily activities causes a loss of dependence, functional decline, deconditioning and muscle atrophy, thereby contributing to further falls [50]. In line with our research, the vicious path still applies to older stroke patients and may lead to severe consequences. Performance of daily activity is an essential part of rehabilitation for stroke recovery [51]; when patients adopt an activity-restricted lifestyle, the recovery of limb function is jeopardized, and rehabilitation is impeded, leading to reduced quality of life.

Secondly, depression was a more critical psychological factor related to FOF instead of anxiety, as illustrated by the final model. There were contradicting facts on the effects of anxiety and depressive symptoms on FOF; one study placed anxiety adjacent to FOF and suggested that FOF may arise from general anxiety [52]. A meta-analysis suggested anxiety as a risk factor for FOF [16]. But a cross-sectional survey found that depression was correlated with the FOF scores, while anxiety was uncorrelated with FOF [53]. A longitudinal study found an independent association between incident FOF and depressive symptoms [54]. Our study supported the relationship between depression and FOF. According to the Chinese Classification and Diagnostic Criteria of Mental Disorders, 3rd Edition (CCMD-3), anxiety symptoms were upwardly hyperactive and characterised mainly by nervousness without a clear object or specific content. Depressive symptoms were inhibited downward and dominated by a depressed state of mind that could range from grumpy or grief [55]. In this sense, depression is more related to the avoidance of activity after a stroke due to low mood or motivation, contributing to a bidirectional, mutually reinforcing relationship among depressive symptoms, FOF, and activity restrictions [56]. Our results suggested that compared to anxiety, depression might be a more important factor to consider when tailoring interventions to alleviate FOF for older patients with stroke.

Thirdly, female sex, fall history, and compromised sides were related to FOF, while neither hearing nor visual problems were associated with FOF. It was reported that women showed significantly worse FOF conditions than men [57]; this gender difference still applies to older stroke patients. FOF in women over 60 years of age has been associated with factors such as menopause, which may generate a decrease in bone mineral mass and hormones [58]. The compromised sides had a weak association with FOF, and according to the univariate analysis of Table 1, compromised sides were not related to different levels of FOF; this is a controversial result and needs further study. Consistent with the previous study [59], hearing problems tended to affect fall risk more compared to FOF; this could be reflected during the investigation when patients with serious visual and hearing issues denied the presence of FOF. The reason could be that FOF is related to the level of self-imposed danger of falling [60]. When the perceptions of the danger of falling are in line with the actual balance ability, FOF itself will encourage positive, protective changes to behaviour [18]. But due to the impaired sensory systems, older stroke patients are more likely to over/under-estimating the danger of falling; this mismatch between perception and reality may jeopardise the adaptive side of FOF.

### Strengths and limitations

The study has a number of strengths: To date, there is limited evidence about how the FOF theoretical framework is applied to older patients with stroke, and this is the first investigation on the interrelationship of FOF and fall risk together with physiological and psychological constructs using suitable and reliable measurement tools. We used activity restrictions as a direct target outcome rather than measured it via other variables, such as FOF, and the primary constructs of the final FOF model were measured by questionnaires with good validity and reliability. Further, the original framework was expanded and adjusted to be more adaptable to stroke patients, which offers an insightful way to study FOF and falls for future research.

But there are still limitations: some diseases like hypertension and diabetes were not taken into consideration, which may have helped to gain more insight into possible risks of FOF and fall risk. Fall history was measured by falls in the past year, which caused recalling bias, and fall risk was assessed by a self-rated questionnaire; it might be part of the FOF construct; more objective measurement methods should be formulated in future research. Regarding the possession of data collection, we used a convenience sample of older stroke patients; data was collected in outpatient and inpatient departments, so our findings could not be extrapolated to a broader range and more vulnerable populations. Further, this cross-sectional study offers no prospective conclusion about the observed relationship between FOF and fall risk.

## Conclusions

The increased risk of falling in older stroke patients results from a maladaptive FOF affected by depression, anxiety, past falls, poor balance, and limited activity. Our results support the previous finding about the FOF theoretical framework [13], suggesting more significant attention to FOF during stroke recovery and fall prevention. A multifaced intervention program encompassing physiological and psychological factors should be designed. Besides, the distinction between objective reality and subjective perception of fall risk requires further studies; qualitative research may help explain the formation of the difference. In addition, our results show that psychological factors, female gender, and fall history should be considered when tailoring interventions for older stroke patients to address falls.

## Data Availability

The datasets used during the study are available from the author upon reasonable request. Please get in touch with Yuanyuan Chen through e-mail: a17812207837@163.com.

## Abbreviations

FOF: Fear of Falling
BMI: Body mass index
FES-I: The Falls Efficacy Scale International
FRQ: The Self-Rated Fall Risk Questionnaire
FSBT: The Four-Stage Balance Test
GAD-7: The 7-item Generalized Anxiety Disorder scale
GDS-15: The 15-item Geriatric Depression Scale
MMSE: Mini-Mental State Examination
IPAQ-LF: The long-form International Physical Activity Questionnaire
SD: Standard deviation
